# Intra-articular injection of synovium-derived mesenchymal stem cells and hyaluronic acid promote regeneration of massive cartilage defects in rabbits

**DOI:** 10.5195/cajgh.2013.97

**Published:** 2014-01-24

**Authors:** Vyacheslav Ogay, Miras Karzhauov, Ainur Mukhambetova, Eric Raimagambetov, Nurlan Batpenov

**Affiliations:** 1 Laboratory of Stem Cells, National Center for Biotechnology, Almaty, Kazakhstan; 2 Scientific and Research Institute of Traumatology and Orthopedics, Almaty, Kazakhstan

**Keywords:** synovium-derived mesenchymal stem cells, cartilage regeneration, hyalunoric acid

## Abstract

**Introduction:**

The purpose of this study was to investigate whether intra-articular injection of synovium-derived mesenchymal stem cells (SD MSCs) with low molecular weight hyaluronic acid (HA) could promote regeneration of massive cartilage in rabbits.

**Material and methods:**

The SD MSCs were harvested from the knees of 10 Flemish giant rabbits, expanded in culture, and characterized. A reproducible 4-mm cylindrical defect was created in the intercondylar groove area using a kit for the mosaic chondroplasty of femoral condyle COR (De Puy, Mitek). The defect was made within the cartilage layer without destruction of subchondral bone. Two weeks after the cartilage defect, SD MSCs (2 × 106 cell/0.15 ml) were suspended in 0.5% low molecular weight HA (0.15 ml) and injected into the left knee, and HA solution (0.30 ml) alone was placed into the right knee. Cartilage regeneration in the experimental and control groups were evaluated by macroscopically and histologically at 10, 30, and 60 days.

**Results:**

On day 10, after intra-articular injection of SD MSCs, we observed an early process of cartilage regeneration in the defect area. Histological studies revealed that cartilage defect was covered by a thin layer of spindle-shaped undifferentiated cells and proliferated chodroblasts. In contrast, an injection of HA did not induce reparation of cartilage in the defect area. At 30 days, macroscopic observation showed that the size of cartilage defect after SD MSC injection was significantly smaller than after HA injection. Histological score was also better in the MSC-treated intercondylar defect. At 60 days after MSC treatment, cartilage defect was nearly nonexistent and looked similar to an intact cartilage.

**Conclusion:**

Thus, intra-articular injection of SD MSCs can adhere to the defect in the intercondylar area, and promote cartilage regeneration in rabbits.

